# Invisible, but how? The depth of unconscious processing as inferred from different suppression techniques

**DOI:** 10.3389/fpsyg.2014.01117

**Published:** 2014-10-01

**Authors:** Julien Dubois, Nathan Faivre

**Affiliations:** Division of Biology, California Institute of TechnologyPasadena, CA, USA

**Keywords:** unconscious processing, Continuous Flash Suppression (CFS), backward masking, visual crowding, invisibility, measures of consciousness, interocular suppression, psychophysical magic

To what level are invisible stimuli processed by the brain in the absence of conscious awareness? Taking stock of the evidence to this day, it is widely accepted that simple visual properties of invisible stimuli are processed; however, the existence of higher-level unconscious processing (e.g., involving semantic or executive functions) remains a matter of debate. After several years of research in the field of unconscious processing, we became aware of a number of methodological aspects which need to be controlled carefully to help resolve discrepant findings in the literature. These aspects relate to: (1) when and how visibility is assessed; (2) when and how unconscious processing is assessed; (3) whether spatiotemporal attention is directed or, at least, measured; (4) whether adequate control conditions are used to rule out alternate explanations; (5) whether the studies are sufficiently powered and account for individual differences. Yet even when these aspects are carefully controlled, there may be, and probably are, some inherent differences in the amount of information let through by the different invisibility-inducing techniques (the “psychophysical magic” arsenal, Kim and Blake, 2005). We launched this Research Topic to foster investigations into these inherent differences (note previous attempts, Breitmeyer et al., 2004; Almeida et al., 2008; Kanai et al., 2010; Faivre et al., 2012).

The articles in this issue span various aspects of the research question that we put out to the community.

Perhaps the best starting point for a thorough introduction to the field of unconscious processing is the contribution by Landry et al. (2014): they review the different techniques used to prevent visual awareness, emphasizing the distinction between subliminal vs. preconscious processing, and find difficulty in reconciling the variety of techniques and results in the literature. They advocate the use of hypnosis, a top-down approach that can induce both perceptual and attentional failures, as a way to bridge the existing gap in the literature.

Continuous Flash Suppression (CFS) (Tsuchiya and Koch, 2005; Tsuchiya et al., 2006) consists in presenting a low contrast target stimulus to one eye while flashing a stream of high contrast stimuli to the other eye, resulting in strong interocular suppression. This rather young technique (as compared to masking or binocular rivalry) has boosted the field of unconscious processing in the past 10 years, allowing researchers to render stimuli invisible for seconds at a time in a very robust fashion. A slight but significant twist referred to as “breaking Continuous Flash Suppression” (bCFS) (Jiang et al., 2007), which consists in comparing the time it takes for different stimuli to break suppression, has been in the spotlight recently. Using bCFS, researchers have claimed that many high-level properties of invisible stimuli are processed unconsciously, which clashes with classical results from binocular rivalry and CFS itself. Yang et al. (2014) offer an insightful review of behavioral CFS and bCFS findings. Though they conclude that the emerging picture is that extensive processing can occur under CFS, they caution against many common issues in the use of (b)CFS. Gayet et al. (2014) focus exclusively on bCFS, and review the 30 studies published to date using this technique. They reject most conclusions of high-level processing, arguing that low-level mechanisms could account for the data. In a similar vein (Stein and Sterzer, 2014) argue that bCFS is not a valid measure of consciousness in its current implementation, and propose some modifications to the paradigm. Taking a step back from the controversies about bCFS (Sterzer et al., 2014) review neural processing under interocular suppression. They conclude that the literature currently presents highly heterogeneous findings regarding which structures are involved in processing suppressed stimuli and which stimuli can be processed; they emphasize the importance of controlling the depth of suppression in future studies, and advocate the use of online, continuous measures that capture the functional relevance of brain signals related to the processing of invisible stimuli. Finally (Faivre et al., 2014) compare published findings in the CFS and visual crowding literatures, as two methods designed to induce sustained invisibility. They conclude that the literature does not yet provide a coherent picture on the extent of processing under each of the two methods.

Taken together, these six review and opinion articles paint an accurate picture of the current landscape and controversies around unconscious processing. In addition, we received six original research articles.

A crucial choice in unconscious processing studies is that of the measure to establish invisibility, and there is still considerable debate on what this measure should be. This is illustrated in a contribution by Herzog et al. (2014), in which the very notion of invisibility is challenged, and reformulated in terms of a purposeful interpretation (i.e., spatiotemporal grouping) of incoming stimuli by the brain. In this view, the features of incoming stimuli are always visible and available to the brain, it is just a matter of how they are interpreted. Based on a more traditional definition of invisibility (Sandberg et al., 2014) compare exclusion tasks, in which participants are asked to solve an experimental task without using information from the invisible stimulus, with subjective visibility measures, in which participants are asked to indicate their subjective experience on a perceptual awareness scale (PAS). They find that exclusion tasks may in fact be a less sensitive and exhaustive measure of awareness than the PAS.

Another important choice in unconscious processing studies is what exact parameters should be used, within a given technique, to create the conditions of invisibility that one wishes to work with. Kaunitz et al. (2014) investigate the role of various parameters (onset time of the stimulus with respect to the onset of the masks, mask frequency, number of masks, duration of the stimulus) for the masking of a brief target stimulus with CFS. They notably find that showing a number of masks before the target does not increase suppression depth, and that a higher temporal frequency of Mondrian presentation results in deeper suppression. Their study demonstrates the importance of small, often overlooked, experimental details which can have an impact on the outcome of different studies and prevent comparison of results.

Finally, we received three studies comparing the extent of unconscious processing under different suppression techniques, either at a behavioral or at a neural level. Peremen and Lamy (2014) compare priming for directional arrow stimuli under metacontrast masking and CFS, in two separate experiments. They find that priming occurs with metacontrast masking but is abolished when stimuli are rendered invisible with CFS. They use each technique as it is optimally implemented, meaning that a number of experimental factors differ between the two experiments (as in Almeida et al., 2008, 2010). Izatt et al. (2014) also chose to compare masking and interocular suppression, but with a fame priming paradigm using invisible faces. The authors take great care to equate most experimental parameters and randomly present both techniques within the same experiment, such that the subjects are completely unaware of which is used on any given trial. Under these conditions, the authors do not find significant differences in the processing of faces rendered invisible by the two techniques, but they generally observe that priming effects are larger with masking than interocular suppression. Last (Fogelson et al., 2014) render a small set of four faces and four tools invisible using two techniques that allow sustained invisibility: CFS, and the less common chromatic flicker fusion (CFF). They concurrently record fMRI and, under the same condition of invisibility, attempt to decode the category of the invisible stimuli throughout the brain. They find that informative regions differ between the two techniques.

These contributions bring valuable insights into the question that we set out to address when we launched this Research Topic. This is a strong starting point to an in-depth systematic comparison of measures and techniques in the study of unconscious vision. We sincerely hope that the readers will find this collection of articles as stimulating and thought-provoking as we do, and that they will apply some of the many wise recommendations interspersed throughout to their own line of research.

## Conflict of interest statement

The authors declare that the research was conducted in the absence of any commercial or financial relationships that could be construed as a potential conflict of interest.
